# Synergistic efficacy of repetitive peripheral magnetic stimulation on central intermittent theta burst stimulation for upper limb function in patients with stroke: a double-blinded, randomized controlled trial

**DOI:** 10.1186/s12984-024-01341-w

**Published:** 2024-04-08

**Authors:** Chi-Shou Chang, Chia-Ling Chen, Rou-Shayn Chen, Hsieh-Ching Chen, Chung-Yao Chen, Chia-Ying Chung, Katie Pei-Hsuan Wu, Ching-Yi Wu, Keh-chung Lin

**Affiliations:** 1 Department of Diagnostic Radiology, Chang Gung Memorial Hospital, Linkou, Taiwan; 2 Department of Medicine, College of Medicine, Chang Gung University, Taoyuan City, Taiwan; 3 Department of Physical Medicine and Rehabilitation, Chang Gung Memorial Hospital, Linkou, Taiwan; 4 Graduate Institute of Early Intervention, Chang Gung University, Taoyuan City, Taiwan; 5 Department of Physical Medicine and Rehabilitation, Chang Gung Memorial Hospital, Xiamen, China; 6 Neuroscience Research Center, Department of Neurology, Chang Gung Memorial Hospital, Linkou, Taiwan; 7 Institute of Cognitive Neuroscience, National Central University, Taoyuan, Taiwan; 8 Department of Industrial Engineering and Management, National Taipei University of Technology, Taipei, Taiwan; 9 Department of Physical Medicine and Rehabilitation, Chang Gung Memorial Hospital, Keelung, Taiwan; 10 School of Chinese Medicine, College of Medicine, Chang Gung University, Taoyuan, Taiwan; 11 Department of Occupational Therapy, College of Medicine, Chang Gung University, Taoyuan, Taiwan; 12 School of Occupational Therapy, College of Medicine, National Taiwan University, Taipei, Taiwan; 13 Division of Occupational Therapy, Department of Physical Medicine and Rehabilitation, National Taiwan University Hospital, 17, F4, Xu-Zhou Road, Taipei, Taiwan

**Keywords:** Theta burst stimulation, Peripheral magnetic stimulation, Stroke, Upper limb, Motor function, Rehabilitation, Participation

## Abstract

**Background:**

Non-invasive techniques such as central intermittent theta burst stimulation (iTBS) and repetitive peripheral magnetic stimulation (rPMS) have shown promise in improving motor function for patients with stroke. However, the combined efficacy of rPMS and central iTBS has not been extensively studied. This randomized controlled trial aimed to investigate the synergistic effects of rPMS and central iTBS in patients with stroke.

**Method:**

In this study, 28 stroke patients were randomly allocated to receive either 1200 pulses of real or sham rPMS on the radial nerve of the affected limb, followed by 1200 pulses of central iTBS on the ipsilesional hemisphere. The patients received the intervention for 10 sessions over two weeks. The primary outcome measures were the Fugl-Meyer Assessment-Upper Extremity (FMA-UE) and the Action Research Arm Test (ARAT). Secondary outcomes for activities and participation included the Functional Independence Measure-Selfcare (FIM-Selfcare) and the Stroke Impact Scale (SIS). The outcome measures were assessed before and after the intervention.

**Results:**

Both groups showed significant improvement in FMA-UE and FIM-Selfcare after the intervention (*p* < 0.05). Only the rPMS + iTBS group had significant improvement in ARAT-Grasp and SIS-Strength and activity of daily living (*p* < 0.05). However, the change scores in all outcome measures did not differ between two groups.

**Conclusions:**

Overall, the study’s findings suggest that rPMS may have a synergistic effect on central iTBS to improve grasp function and participation. In conclusion, these findings highlight the potential of rPMS as an adjuvant therapy for central iTBS in stroke rehabilitation. Further large-scale studies are needed to fully explore the synergistic effects of rPMS on central iTBS.

**Trial registration:**

This trial was registered under ClinicalTrials.gov ID No.NCT04265365, retrospectively registered, on February 11, 2020.

**Supplementary Information:**

The online version contains supplementary material available at 10.1186/s12984-024-01341-w.

## Background

Stroke is a leading cause of death and disability worldwide, with impaired upper limb motor function being a common outcome for stroke survivors. According to the Global Burden of stroke 2019, stroke had become the second most common causes of death (11.6% of all deaths [95% uncertainty interval, 10.8–12.2%]) and the third most common causes of disability (5.7% of disability-adjusted life years from all causes [95% uncertainty interval, 5.1–6.2]) in the world [1]. Among people experiencing stroke episodes, impaired motor function of upper extremities often had adverse effects on the daily activities [2] and participation [3]. In 70% of stroke patients, upper limb involvement was responsible for long-term impairment of daily function and activities [4, 5].

Even with traditional neurorehabilitation programs, approximately 50–60% of stroke patients still experience chronic motor limitations [6]. To address this, non-invasive brain stimulation such as central theta burst stimulation (TBS), a novel form of repetitive transcranial magnetic stimulation (rTMS), have been used to treat these patients [7]. Central TBS has been found to have persistent effects on motor evoked potentials (MEPs) [8, 9]. The bimodal balance-recovery model has been proposed as the underlying mechanism for central rTMS [10]. This model combined the concepts of interhemispheric competition and vicariation effects of the intact hemisphere in patients with stroke [10]. The hypothesis posited that there was a reduction in cortical excitability within the impaired hemisphere, accompanied by an increase in transcallosal inhibitory signaling originating from the intact hemisphere [10]. To facilitate cortical excitability in the impaired hemisphere, intermittent TBS (iTBS) is applied, while continuous TBS (cTBS) is utilized to reduce transcallosal inhibitory signals in the intact hemisphere [11]. A recent meta-analysis has shown that iTBS outperforms cTBS in terms of promoting upper limb motor recovery in stroke patients [12]. Therefore, iTBS was selected for this study.

Repetitive peripheral magnetic stimulation (rPMS) is another non-invasive brain stimulation technique that targets the peripheral motor nerve through both direct and indirect activation [13–15]. The transmission of direct activation occurred through the sensorimotor nerve, whereas indirect activation was facilitated by the mechanoreceptor nerve [13–15]. It has been hypothesized that rPMS could induce neuroplasticity and cortical reorganization [13–15]. Prior research has demonstrated increased motor evoked potential (MEP) amplitudes in the upper limb following rPMS application [16–19]. One study demonstrated the potential of rPMS to enhance distal motor function [20], and another showed its effectiveness in improving proximal muscle strength in early subacute stroke patients [21]. Recent studies further underscore the positive impact of rPMS on upper motor function assessed by Fugl-Meyer Assessment (FMA) during the subacute and acute phases of stroke [21, 22]. Furthermore, FMA-Upper Extremity (FMA-UE) includes proximal and distal domain [23, 24]. Considering that most patients with stroke suffered from flexor spasticity in the upper limb, which limited their ability to open hands for object manipulation. Thus, we chose the radial nerve for the delivery of rPMS, which is essential for the recovery of skilled hand prehension [25].

To date, the majority of studies have focused on the effects of integrating rPMS with rehabilitation programs [20–22] for patients with stroke. Currently, one study showed that central rTMS combined with rPMS altered cerebellar and frontoparietal cortical activity via functional magnetic images [26]. One study combined rTMS with rPMS to improved patient’s spasticity and motor function [27]. While the individual benefits of central rTMS and rPMS have been documented in previous studies [26, 27], our rationale for combining them is based on emerging evidence that rPMS can modulate motor cortical excitability in the central nervous system [13–15]. Furthermore, the iTBS was proved to have more enduring effects than the conventional rTMS [2, 28]. This concept is still relatively novel, and no studies have explored the synergistic effects of central iTBS when combined with peripheral rPMS. Therefore, we hypothesized that applying rPMS to the radial nerve might enhance the effectiveness of central iTBS over the primary motor cortex, leading to improvement in motor function, activities, and participation. This is the first randomized controlled trial investigating the synergistic efficacy of rPMS on central iTBS in treating upper limb dysfunction in patients with stroke.

## Method

### Participants

Between 2019 and 2021, we recruited 28 stroke patients from the rehabilitation department of Chang Gung Memorial Hospital. We screened 557 patients, but excluded 525 patients. Four patients declined to participate, leaving us with a total of 28 participants. We randomly assigned them to either the rPMS + iTBS group (*n* = 14) or the sham rPMS + iTBS group (*n* = 14). The inclusion criteria for the study were as follows: (1) aged between 20 and 80 years; (2) ischemic or hemorrhagic stroke for the first time; (3) unilateral cerebral stroke with hemiplegia or hemiparesis; and (4) subacute (between 7 days and 6 months since onset) or chronic (more than 6 months since onset) stages of stroke [29]. The exclusion criteria were as follows: (1) stroke at brainstem or cerebellum; (2) progressive neurodegenerative diseases; (3) history of epilepsy; (4) medical histories of aneurysm and cerebral arteriovenous malformation; (5) patients with active medical problems; (6) active psychiatric disorders; (7) severe cognitive and language impairment; (8) metal implants such as pacemakers, head metal implants, and aneurysm clips; (9) Botox injections in six months; and (10) patients who are pregnant or who are probable pregnant. All patients signed the informed consent prior to the enrollment. The study protocol was executed in accordance with the Declaration of Helsinki and was approved by Chang Gung medical foundation institutional review board. This trial is registered under ClinicalTrials.gov ID No. NCT04265365.

### Study design

**Fig. 1:**
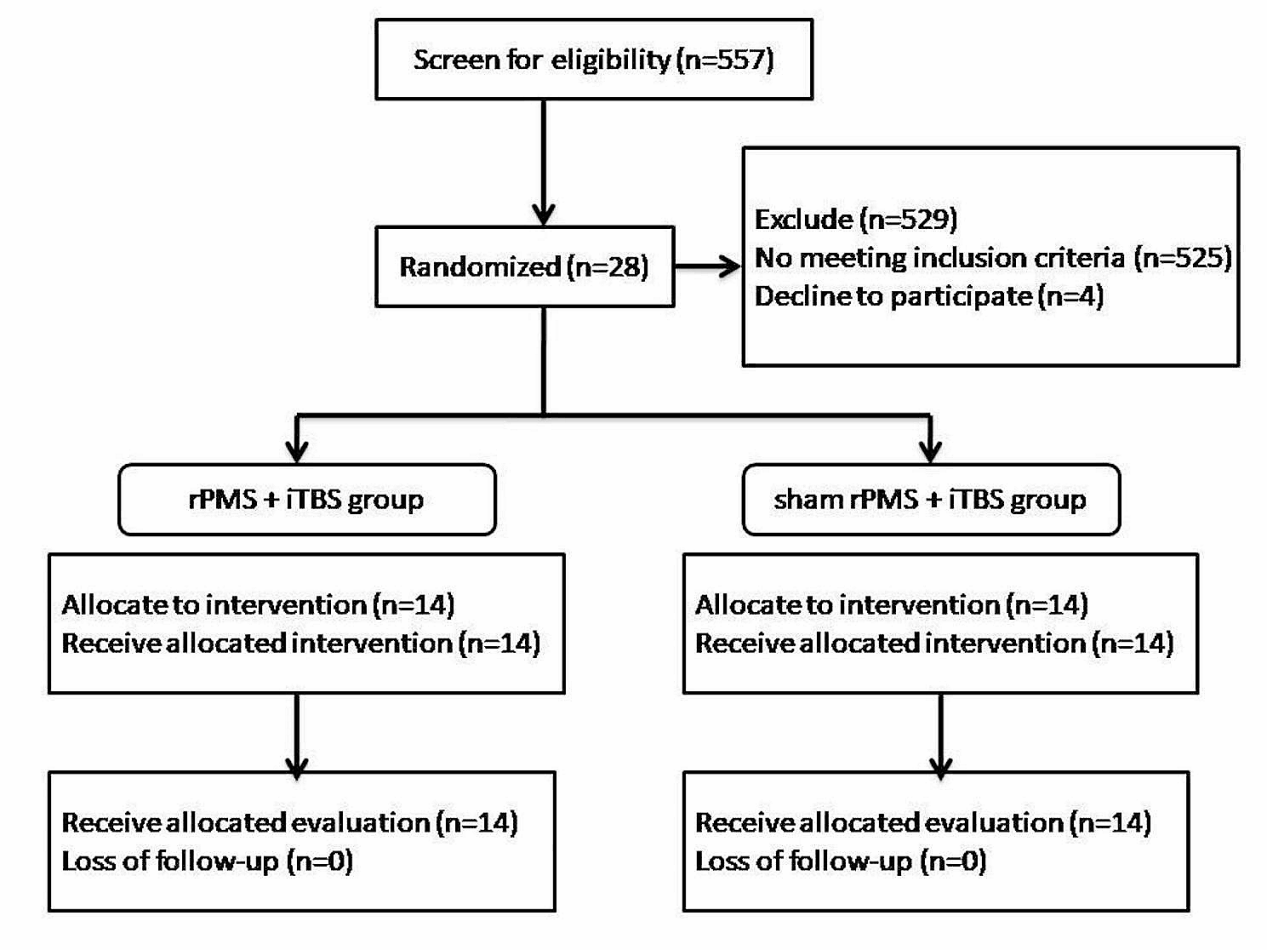
Flow diagram of recruitment and randomized allocation

**Fig. 2:**
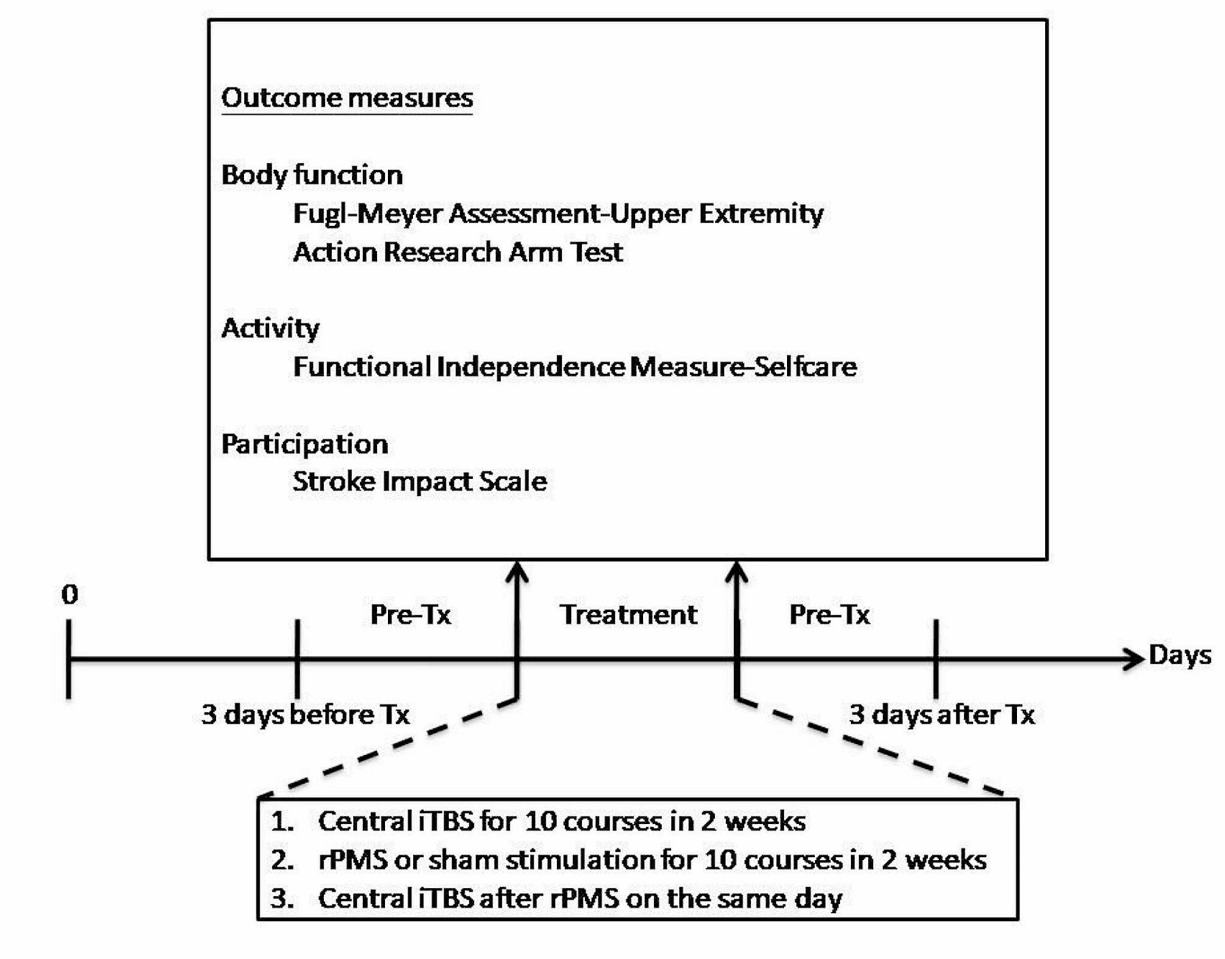
Experimental protocol

The study was a prospective double-blinded, randomized, controlled trial. Various measures or restriction were used to ensure intervention and assessment accuracy and consistency. The severity of stroke was stratified according to Brunnström stage [30, 31] before randomly allocating the twenty-eight patients into two groups on the website (https://www.randomizer.org/) because the Brunnström stage is a quick and convenient bedside evaluation method to assess a patient’s recovery stage. The clinical data were also recorded. Figures 1 and 2 demonstrated the randomized allocation and experimental design, respectively. The patients were asked to relax 5 min before, during, and 5 min after the stimulation to avoid the effects of physical activities on central iTBS. The patients received 10 courses of central iTBS with real or sham rPMS on consecutive working days for 2 weeks. The rPMS was delivered before central iTBS. Outcome measures were performed 3 days before intervention and after intervention. The evaluators were well trained to administer outcome measures prior to the project after passing competency and reliability test. At least two professional evaluators blinded to the group assignment performed the outcome measure and, both intra-rater and inter-rater reliability should be recorded. The intra-rater/inter-rater reliability of the Action Research Arm Test (ARAT) and FMA-UE was 0.986/0.998 and 0.984/0.992, respectively. The intra-rater reliability of the Functional independence measure-Self care (FIM-Self care) and Stroke impact scale (SIS) were 0.994, 0.956, respectively. Besides magnetic stimulation, patients received comprehensive rehabilitation programs, including physical therapy (60 min) and occupational therapy (60 min), both administered five times a week. Additionally, specialized speech-language therapy was provided for patients exhibiting symptoms related to speech and swallowing issues.

### Participant selection and positioning

Participants were carefully screened based on inclusion/exclusion criteria. This involved assessing upper limb dysfunction severity via the Brunnström stage, ensuring medical stability, and verifying their comprehension and participation capability. Participants were consistently positioned, with forearms resting on a desk during rPMS and central iTBS administration to minimize variability.

### Randomization and blinding

To ensure the integrity of the blinding process, participants were randomly assigned to either the rPMS + iTBS group or the sham rPMS + iTBS group through the (https://www.randomizer.org/) website. The generated sequence was concealed from both the researchers and the participants until the allocation process was completed, maintaining the integrity of the randomization process. Patients were then assigned to their respective groups according to the generated sequence. An independent researcher not directly involved in assessments or interventions carried out the allocation. Importantly, the operators responsible for administering the stimulation and the evaluators conducting the outcome measurements were separate individuals, and they were unaware of each other’s roles. Furthermore, the evaluators remained blinded to the patients’ group assignments throughout the study, enhancing the study’s double-blind nature. Additionally, patients were kept unaware of the specific treatment conditions, further ensuring the blinding of the intervention.

### Standardization of assessments and interventions

All individuals, including evaluators and operators, underwent comprehensive training and competence tests to ensure their qualifications and expertise. Evaluators were trained for assessment accuracy, and supervised by the principal investigator and a senior certified occupational therapist. Operators were trained and supervised by the principal investigator and a senior neurologist to ensure standardized equipment operation.

### Determination of AMT and RMT

The study used the TMS protocol to evaluate the Active Motor Threshold (AMT) and Resting Motor Threshold (RMT ). The MagProX100 package (Magventure, USA)was used for magnetic stimulation with a figure-of-eight coil (outer diameter of each wing 7.5 cm). Silver/silver chloride (Ag/AgCl) disc electrodes were attached to the first dorsal interosseous muscles (FDI) of the affected limb. The coil was positioned tangentially to the scalp over the motor area of the affected limb in the optimal position for activating the FDI. The handle was held pointing backward and laterally at an angle of 45° to the sagittal plane to generate a posterior-to-anterior current flow in the brain. The point at the scalp that induced the maximum MEP was identified as the motor hot spot [32]. If the MEPs could not be induced, we would identify the motor hot spot from the mirror site from unaffected hemisphere [32]. The MEP was recorded using a Multifunctional Response and Stimulus Device (BioPAC Inc, USA). The patients were seated in a comfortable chair with their forearm in a pronated position on the desk. They were instructed to remain relaxed throughout the procedure. The RMT was defined as the minimal intensity of TMS that induced an MEP equal to the 50 uV peak-to-peak amplitude when the FDI was relaxed in at least 5 of 10 consecutive trials. The AMT was defined as the intensity of TMS that induced an MEP equal to the 200 uV peak-to-peak amplitude when the FDI slightly contracted in at least 5 of 10 consecutive trials (10–20% of maximum contraction).

### Central iTBS and rPMS protocol

During the study, we administered iTBS to either the affected primary motor cortex as central iTBS or to the radial nerve as rPMS. To administer central iTBS, we used a handheld figure-of-eight coil at the motor hot spot of the first dorsal interosseous (FDI) muscle, while for rPMS, we applied the coil at the radial groove.

As for central iTBS, we administered iTBS to the affected primary motor cortex. The true stimulation was given at 80% of AMT or 70% of RMT.

As for rPMS, the stimulation intensity for real rPMS was individually adjusted for each participant to achieve a joint movement resulting from muscle contraction of extensor carpi radialis muscle. In contrast, sham stimulation was set at a low-intensity level at 5% of the maximal stimulator output [33], which did not induce the MEP in the extensor carpi radialis muscle. The participants receiving sham stimulation were still able to perceive the noise, leading them to maintain the belief that they were indeed receiving the stimulation throughout the study. Besides, participants were kept separate from each other and were unaware of the group assignments. Each session of iTBS comprised 20 rounds of stimulation. Each round consisted of a 2-second burst at 5 Hz followed by an 8-second period of rest. Each burst contained 3 pulses at 50 Hz, resulting in a total of 600 pulses per session, which lasted 200 s. We administered 2 sessions of iTBS for central iTBS and rPMS, with a 10-minute break between sessions. The stimulation was conducted at the same time each day for five consecutive days per week for two weeks.

### Outcome measures

The primary outcomes were the improvement in motor function, as reflected by the scores on the FMA-UE and the Action Research Arm Test (ARAT). The FMA-UE is a performance-based scale used to evaluate motor function of upper limbs in patients with stroke [23]. In contrast, the ARAT is composed of 19 items divided into four subsections, including grasp, grip, pinch, and gross movement [24].

Secondary outcomes focused on improvement in activities and participation. Activities were assessed using the self-care domain of the Functional Independence Measure (FIM). The self-care domain was selected for this study because it comprises six items specifically associated with activities of the upper limb [34].

Participation was evaluated using the Stroke impact scale (SIS), a self-report assessment of disability and quality of life after stroke [35]. The SIS includes eight domains: strength, hand function, activities of daily living (ADL), mobility, communication, emotion, memory and thinking, and participation. The scale comprises 59 items that are rated on a 5-point scale, with a score of 5 representing the best performance in participation.

### Statistical analysis

The data was processed by SPSS version 21.0 for Windows (SPSS Inc., Chicago, IL, USA). Shapiro–Wilk tests were adopted to confirm the assumptions of normal distribution, and only total SIS, some SIS domains, and FIM-Self care met the criteria. Therefore, we used nonparametric method to analyze the data. The baseline of outcomes and demographic of clinical characteristics were analyzed under Chi-square test or Fisher’s exact test for categorical variables and an Independent t-test for continuous data. Wilcoxon signed-rank test was applied to determine whether each group had significant improvement after the intervention. Analysis of covariance was adapted to test whether the rPMS + iTBS group showed greater improvement than the sham rPMS + iTBS group after treatment. We defined pre-treatment performance and baseline differences (sex, and Mini Mental Status Examination (MMSE)) as the covariates, with group as the independent variable, and post-treatment performance as the dependent variable. We conduct power analysis for repeated measures design using G*Power software (G*Power 3.1.9.7) to estimate our sample size requirement [36]. We found that a total of 27 participants will be required for effect size of 0.5 with a power of 0.8 and type I error of 0.05. Thus, we recruited 28 participants, resulting in 14 participants for each group. The significance was pinpointed at 0.05 (one-tailed) and, we adopted t-distribution to determine a 95% confidence interval (95% CI) for small sample size (*n* < 30).

### Result

The demographic and clinical data did not differ in age, stroke side, stroke stage, stroke location, stroke type, aphasia, MMSE, NIHSS, modified Ashworth scale, and Brunnström stage except sex (Table 1). The baseline of outcome measures did not differ between groups (Table 2). All patients well tolerated the intervention without significant adverse effects in the study.

**Table 1:** Demographic and clinical characteristics

	rPMS + iTBS	sham rPMS + iTBS	*p*-value
Age	51.4 ± 12.1	55.6 ± 10.3	0.326^b^
Sex Male Female			0.021^a^
	4 (28.6%)	11 (78.6%)	
	10 (71.4%)	3 (21.4%)	
Stroke stage			0.705^a^
Subacute	8 57.1%)	7 (50.0%)	
Chronic	6 (42.9%)	7 (50.0%)	
Stroke side Left Right			0.705^a^
	6 (42.9%)	7 (50.0%)	
	8 (57.1%)	7 (50.0%)	
Stroke location Subcortical Cortical Combined			0.838^a^
	7 (50.0%)	9 (64.3%)	
	6 (42.9%)	4 (28.6%)	
	1 (7.1%)	1 (7.1%)	
Stroke type Hemorrhage Infarction			0.695^a^
	8 (57.1%)	10 (71.4%)	
	6 (42.9%)	4 (28.6%)	
Aphasia Yes No			1.000 ^a^
	2 (14.2%)	2 (14.3%)	
	12 (85.7%)	12 (85.7%)	
MMSE	23.4 ± 8.5	27.8 ± 2.8	0.075^b^
NIHSS	9.9 ± 6.5	8.6 ± 5.3	0.594^b^
MAS	1.1 ± 0.8	1.1 ± 0.7	0.432^b^
Brunnström-UEp	3.5 ± 1.1	3.5 ± 0.9	1.000^b^
Brunnström-UEd	3.3 ± 1.1	3.6 ± 1.4	0.474^b^

Data are presented as mean ± standard deviation or number (%);^a^Chi-square test or Fisher’s exact test; ^b^Independentt-test; MMSE: Mini-MentalState Exam; NIHSS: National Institutes of Health Stroke Scale; MAS: Modified Ashworth Scale; Brunnström-UEp: Brunnström Stage-proximal; Upper Extremity; Brunnström-UEd: Brunnström Stage-distal Upper Extremity

**Table 2:** Baseline of outcome measure

	rPMS + iTBS	Sham rPMS + iTBS	*p*-value
FMA-UE	28.6 ± 21.3	33.4 ± 19.7	0.542
FIM-Self care	4.2 ± 1.6	4.7 ± 1.4	0.407
ARAT	19.1 ± 20.9	21.8 ± 23.8	0.758
ARAT-GM	4.1 ± 2.9	5.0 ± 3.9	0.484
ARAT-Grasp	6.0 ± 7.3	6.6 ± 7.9	0.825
ARAT-Grip	4.3 ± 4.8	4.8 ± 5.6	0.801
ARAT-Pinch	4.8 ± 7.1	5.4 ± 7.6	0.838
SIS	53.2 ± 11.0	58.9 ± 14.7	0.252
SIS-S1	29.0 ± 13.8	33.0 ± 14.0	0.450
SIS-S2	82.7 ± 16.3	85.5 ± 19.0	0.678
SIS-S3	52.6 ± 12.0	55.0 ± 11.2	0.593
SIS-S4	83.4 ± 27.8	95.2 ± 7.9	0.150
SIS-S5	51.3 ± 12.2	54.6 ± 26.0	0.664
SIS-S6	50.2 ± 25.6	57.7 ± 30.7	0.487
SIS-S7	23.9 ± 27.8	26.4 ± 35.6	0.838
SIS-S8	37.7 ± 21.2	48.4 ± 19.8	0.179

Data are presented as mean ± standard deviation; ARAT: Action Research Arm Test; GM: Gross motor; FIM: Functional Independence Measure; FMA-UE: Fugl-Meyer Assessment-Upper Extremity; SIS: Stroke Impact Scale, S1: Strength; S2: Memory and Thinking; S3: Emotion; S4: Communication; S5: Activity of Daily Living; S6: Mobility; S7: Hand Function; S8: Participation

### Primary outcomes

#### Motor function

In FMA-UE, both groups had significant improvement after the intervention (rPMS group: *p* = 0.019; sham group: *p* = 0.025) (Table 3). However, the change scores did not differ in FMA-UE between groups.

**Table 3:** Descriptive and inferential statistics of primary outcome measures

Variables	rPMS + central iTBS					sham rPMS + central iTBS					
	Pre-Tx		Post-Tx		p (WSRT)	Pre-Tx		Post-Tx		p (WSRT)	p (ANCOVA)
	mean ± SD	95% CI	mean ± SD	95% CI		mean ± SD	95% CI	mean ± SD	95% CI		
FMA-UE	28.6 ± 21.2	16.4 ~ 40.9	37.4 ± 20.4	19.9 ~ 44.3	0.019*	33.4 ± 19.7	22.1 ~ 44.8	37.1 ± 19.5	25.9 ~ 48.4	0.025*	0.402
ARAT	19.1 ± 20.9	7.1 ~ 31.2	26.2 ± 23.8	9.7 ~ 36.8	0.062	21.8 ± 23.8	8.1 ~ 35.5	23.6 ± 20.3	11.9 ~ 35.4	0.345	0.174
GM	4.1 ± 2.9	2.4 ~ 5.8	5.4 ± 3.7	2.8 ~ 6.5	0.102	5.0 ± 3.9	2.7 ~ 7.3	4.9 ± 3.1	3.1 ~ 6.7	0.952	0.082
Grasp	6.0 ± 7.3	1.8 ~ 10.2	8.9 ± 8.1	3.1 ~ 12.2	0.013*	6.6 ± 7.9	2.1 ~ 11.2	8.0 ± 6.9	4.0 ~ 12.0	0.107	0.358
Grip	4.3 ± 4.8	1.5 ~ 7.0	5.7 ± 5.6	2.1 ~ 8.5	0.125	4.8 ± 5.6	1.6 ~ 8.0	5.6 ± 4.7	2.9 ~ 8.4	0.201	0.460
Pinch	4.8 ± 7.1	0.7 ~ 8.9	6.1 ± 7.2	1.3 ~ 10.0	0.276	5.4 ± 7.6	1.0 ~ 9.8	5.1 ± 6.7	1.2 ~ 9.0	0.750	0.088

SD: standard deviation, CI: confidence interval, ANCOVA: Analysis of Covariance, WSRT: Wilcoxon signed‑rank tests; t-distribution was used to compute a 95% CI. Degrees of freedom = 27; FMA-UE: Fugl-Meyer Assessment-Upper Extremity, ARAT: Action Research Arm Test, GM: Gross Movement; ^*^p < 0.05

In total ARAT, both groups did not achieve significant improvement after the intervention (Table 3). In Grasp domain of ARAT, only rPMS + iTBS group attained significant improvement. (Grasp domain: rPMS: *p* = 0.013; sham group: *p* = 0.107). The change scores did not differ between groups in total ARAT and the four domains of ARAT.

### Secondary outcomes

#### Activity

In FIM-Self care, both groups achieved significant improvement after the intervention (rPMS group: *p* = 0.013; sham group: *p* = 0.011). The change scores did not differ between groups.

### Participation

In total SIS, both groups did not attain significant improvement after the intervention (rPMS group: *p* = 0.064; sham group: *p* = 0.352). The rPMS + iTBS group had significant improvement in Strength and ADL. The rPMS + iTBS group had borderline improvement in Hand Function. The sham rPMS + iTBS group achieved significant improvement in Mobility. (rPMS group: Strength: *p* = 0.019; ADL: *p* = 0.040; Mobility: *p* = 0.346; Hand Function: *p* = 0.050; sham group: Strength: *p* = 0.385; ADL: *p* = 0.430; Mobility: *p* = 0.034; Hand Function: *p* = 0.562). The change scores did not differ in total SIS and the 8 domains of SIS between groups.

**Table 4:** Descriptive and inferential statistics of secondary outcome measures

Variables	rPMS + central iTBS					sham rPMS + central iTBS						
	Pre-Tx		Post-Tx		p (WSRT)	Pre-Tx		Post-Tx		p (WSRT)		p (ANCOVA)
	mean ± SD	95% CI	mean ± SD	95% CI		mean ± SD	95% CI	mean ± SD	95% CI			
FIM-Selfcare	4.2 ± 1.6	3.3 ~ 5.1	5.1 ± 0.9	4.0 ~ 5.8	0.013*	4.7 ± 1.4	3.9 ~ 5.5	5.3 ± 1.3	4.5 ~ 6.0		0.011*	0.217
SIS	53.2 ± 11.0	46.8 ~ 59.5	66.3 ± 10.8	40.0 ~ 66.2	0.064	58.9 ± 14.7	50.4 ~ 67.4	60.4 ± 13.9	52.4 ~ 68.4		0.490	0.065
SIS-S1	29.0 ± 13.8	21.1 ~ 38.0	44.2 ± 19.8	31.5 ~ 42.6	0.019*	33.0 ± 14.0	25.0 ~ 41.1	37.1 ± 20.9	25.0 ~ 49.1		0.385	0.323
SIS-S2	82.7 ± 16.3	73.3 ~ 92.1	88.1 ± 14.1	68.1 ~ 92.2	0.357	85.5 ± 19.0	74.5 ~ 96.4	81.4 ± 16.4	71.9 ~ 90.9		0.260	0.129
SIS-S3	52.6 ± 12.0	45.7 ~ 59.5	65.9 ± 15.8	50.6 ~ 66.0	0.218	55.0 ± 11.2	48.5 ~ 61.4	55.6 ± 13.6	47.7 ~ 63.4		0.959	0.096
SIS-S4	83.4 ± 27.8	67.4 ~ 99.5	95.5 ± 10.2	63.5 ~ 99.8	0.246	95.2 ± 7.9	90.6 ~ 99.7	94.6 ± 6.8	90.7 ~ 98.6		0.573	0.358
SIS-S5	51.3 ± 12.2	44.2 ~ 58.3	63.3 ± 16.4	48.8 ~ 70.2	0.040*	54.6 ± 26.0	39.6 ~ 69.7	57.5 ± 24.5	43.4 ~ 71.7		0.430	0.148
SIS-S6	50.2 ± 25.6	35.4 ~ 65.0	66.1 ± 21.7	40.4 ~ 69.2	0.346	57.7 ± 30.7	40.0 ~ 75.5	65.3 ± 25.2	50.7 ~ 79.8		0.034*	0.464
SIS-S7	23.9 ± 27.8	7.9 ~ 40.0	43.7 ± 33.1	14.9 ~ 56.6	0.050	26.4 ± 35.7	5.9 ~ 47.0	29.6 ± 33.9	10.1 ~ 49.2		0.562	0.222
SIS-S8	37.7 ± 21.2	25.5 ~ 50.0	51.3 ± 20.8	32.0 ~ 50.2	0.421	48.4 ± 19.8	37.0 ~ 59.9	46.7 ± 25.3	32.1 ~ 61.2		0.683	0.252

SD: standard deviation, CI: confidence interval, ANCOVA: Analysis of Covariance, WSRT: Wilcoxon signed‑rank tests; t-distribution was used to compute a 95% CI. Degrees of freedom = 27; FIM: Functional Independence Measure, SIS: Stroke Impact Scale, S1: Strength, S2: Memory and Thinking, S3: Emotion, S4: Communication, S5: Activity of Daily Living, S6: Mobility, S7: Hand Function, S8: Participation, ^*^p < 0.05

## Discussion

This is the first study to investigate the synergistic efficacy of rPMS on central iTBS in enhancing UL function in stroke patients. Both the rPMS + iTBS group and the sham rPMS + iTBS group showed improvement in FMA and FIM after the treatment. However, only the rPMS + iTBS group exhibited additional improvement in the ARAT-Grasp domains and certain domains of SIS. Although there was no significant difference in the change scores of all outcome measures between the two groups, these findings suggest that rPMS may have potential synergistic effects on central iTBS, particularly in improving grasp function and participation, but not in the activities of self-care.

This study showed that rPMS could enhance significant improvement on central iTBS in grasp domains of ARAT. The grasp movement involves the wrist/hand flexion and extension [25]. The extension of wrists and hands was innervated by the radial nerve, which was the target of the rPMS intervention. Although the mechanism of rPMS to facilitate motor function still remained controversial, there were emerging hypotheses believing that rPMS could change sensorimotor nerve and mechanoreceptor nerve [13–15]. The rPMS could directly stimulate the sensorimotor nerve, and the consequent muscle twitching could give another stimulation via mechanoreceptor [13–15]. The afferent proprioceptive inflow to the CNS induced by the rPMS could also stimulate the neuroplasticity in the primary motor cortex and the supplementary motor area [37]. Additionally, central iTBS has the capacity to induce brain homeostasis within the primary cortex [10]. We believed the central iTBS and rPMS could synergistically alter the neuronal circuits via corticospinal tract [37], which made the significant improvement of ARAT-Grasp. Hence, rPMS may exhibit synergistic effects on central iTBS, enhancing cortical reorganization and consequently improving grasp function.

The study demonstrated that rPMS led to significant improvements on central iTBS in SIS domains related to strength and ADL. The improvement in grasp movement by rPMS on central iTBS might be the causes, which further enhanced strength, and ADL. Moreover, a higher percentage of patients in the rPMS + iTBS group achieved the minimal clinically important difference (MCID) in SIS- Hand Function, Strength, and ADL, and the rPMS + iTBS group also had borderline improvement in Hand Function. For example, in Hand Function, 5 patients (35.7%) in the rPMS + iTBS group achieved MCID, while only 3 patients (21.4%) in the sham rPMS + iTBS group did (17.8 points) [38]. In Strength, 6 patients (42.9%) in the rPMS + iTBS group reached MCID, whereas 5 patients (35.7%) in the sham rPMS + iTBS group did (9.2 points) [38]. Finally, in ADL, 8 patients (57.1%) in the rPMS + iTBS group reached MCID, while only 5 patients (35.7%) in the sham rPMS + iTBS group did (5.9 points) [38]. These results suggest that the combination of rPMS may have synergistic effects on cental iTBS in participation.

In this study, both groups showed improvement in the FMA and FIM after the treatment, but the degree of improvement did not differ between the groups. This suggests that the improvement in these two outcome measures was likely due to central iTBS. The bimodal balance-recovery model proposes that central iTBS helps to balance cortical hyper-excitability from the intact hemisphere. Furthermore, central iTBS has been shown to induce long-term potential-like plasticity changes [8, 39, 40] and alter the balance of synaptic endogenous transmitters [41, 42]. Previous studies have also demonstrated that combining central iTBS with conventional neurorehabilitation programs can improve motor function and activities in chronic stroke patients [43–46]. These findings are consistent with the results of this study, which suggest that the improvement in FIM and FMA was primarily due to central iTBS rather than rPMS.

The study has some limitations that should be taken into consideration when interpreting the results. Firstly, the study recruited patients from a rehabilitation ward using convenience sampling, which may limit the generalizability of the findings to other populations. Secondly, the study did not evaluate the long-term effects of the treatment, which may provide important information about the durability of the treatment effects. Thirdly, there were disparities in the sex and MMSE between groups. Previous research has shown that women tend to have a less favorable prognosis than men [47]. Cognitive function can serve as a predictive factor for an individual’s response to motor rehabilitation [48]. Therefore, we included sex and MMSE as covariates in the statistical analysis to reduce the impact of sex and cognition on intervention outcomes. Forth, given the small sample size of our study, it is imperative to conduct further multi-center randomized controlled trials with larger sample sizes for the optimal protocol of the combined use of peripheral and central neuromodulation interventions. Fifth, the study did not delve into the sequential effects of rPMS and central iTBS. Currently, there is no existing literature exploring the possible effects of the sequences when combining rPMS and central iTBS. Further large-scale research is required to elucidate the sequence effect of these two interventions. Despite these limitations, the study findings provide valuable insights into the potential synergistic effects of rPMS and central iTBSin motor function, activities, and participation.

## Conclusion

In conclusion, this study highlights significant findings regarding the potential synergistic efficacy of rPMS when combined with central iTBS. Specifically, our results demonstrate improved grasp function and increased participation in patients with stroke. This study elucidates that the rPMS could be the novel adjuvant therapy for central iTBS in stroke rehabilitation. Moreover, this study addresses an important gap in the field by exploring the synergistic effects of rPMS and central iTBS, shedding light on a new avenue for enhancing stroke recovery. As a primary contribution, our research underscores the potential benefits of combined interventions and sets the stage for future long-term studies aimed at further elucidating the intricacies of rPMS’s effects in conjunction with central iTBS. These findings have the potential to significantly impact the field of stroke rehabilitation and may pave the way for more effective and comprehensive treatment approaches.

### Electronic supplementary material

Below is the link to the electronic supplementary material.

Supplementary Material 1

## Data Availability

The datasets used and/or analyzed during the current study are available from the corresponding author on reasonable request.

## Abbreviations

ADL: activities of daily living
rPMS: Repetitive peripheral magnetic stimulation
AMT: Active Motor Threshold
MEPs: Motor-evoked potentials
RMT: Resting Motor threshold
rTMS: Repetitive transcranial magnetic stimulation
TMS: Transcranial Magnetic Stimulation
ARAT: Action Research Arm Test
GM: gross motor
cTBS: Continuous TBS
FMA-UE: Fugl-Meyer Assessment-Upper Extremity
UE: Upper Extremity
TBS: Theta burst stimulation
iTBS: Intermittent TBS
FIM: Functional Independence Measure
SIS: Stroke Impact Scale
UL: Upper limb

## References

[1] Feigin VL, Stark BA, Johnson CO, Roth GA, Bisignano C, Abady GG, Hamidi S (2021). Global, regional, and national burden of stroke and its risk factors, 1990–2019: a systematic analysis for the global burden of Disease Study 2019. Lancet Neurol.

[2] Ds NL (2005). Factors influencing stroke survivors’ quality of life during subacute recovery. Stroke.

[3] Hartman-Maeir A, Soroker N, Ring H, Avni N, Katz N (2007). Activities, participation and satisfaction one-year post stroke. Disabil Rehabil.

[4] Hankey GJ, Jamrozik K, Broadhurst RJ, Forbes S, Anderson CS (2002). Long-term disability after first-ever stroke and related prognostic factors in the Perth Community Stroke Study, 1989–1990. Stroke.

[5] Barreca S, Wolf SL, Fasoli S, Bohannon R (2003). Treatment interventions for the paretic upper limb of stroke survivors: a critical review. Neurorehabilit Neural Repair.

[6] Hendricks HT, Van Limbeek J, Geurts AC, &Zwarts MJ (2002). Motor recovery after stroke: a systematic review of the literature. Arch Phys Med Rehabil.

[7] Platz T, Rothwell JC (2010). Brain stimulation and brain repair–rTMS: from animal experiment to clinical trials–what do we know?. Restor Neurol Neurosci.

[8] Hernandez RV, Navarro MM, Rodriguez WA, Martinez Jr JL, LeBaron RG (2005). Differences in the magnitude of long-term potentiation produced by theta burst and high frequency stimulation protocols matched in stimulus number. Brain Res Protoc.

[9] Huang YZ, Edwards MJ, Rounis E, Bhatia KP, Rothwell JC (2005). Theta burst stimulation of the human motor cortex. Neuron.

[10] Di Pino G, Pellegrino G, Assenza G, Capone F, Ferreri F, Formica D, Di Lazzaro V (2014). Modulation of brain plasticity in stroke: a novel model for neurorehabilitation. Nat Reviews Neurol.

[11] Sung WH, Wang CP, Chou CL, Chen YC, Chang YC, Tsai PY (2013). Efficacy of coupling inhibitory and facilitatory repetitive transcranial magnetic stimulation to enhance motor recovery in hemiplegic stroke patients. Stroke.

[12] Bai Z, Zhang J, Fong KN (2022). Effects of transcranial magnetic stimulation in modulating cortical excitability in patients with stroke: a systematic review and meta-analysis. J Neuroeng Rehabil.

[13] Struppler A, Binkofski F, Angerer B, Bernhardt M, Spiegel S, Drzezga A, Bartenstein P (2007). A fronto-parietal network is mediating improvement of motor function related to repetitive peripheral magnetic stimulation: a PET-H2O15 study. NeuroImage.

[14] Krause P, Straube A (2008). Peripheral repetitive magnetic stimulation induces intracortical inhibition in healthy subjects. Neurol Res.

[15] Heldmann B, Kerkhoff G, Struppler A, Havel P, Jahn T (2000). Repetitive peripheral magnetic stimulation alleviates tactile extinction. NeuroReport.

[16] Gallasch E, Christova M, Kunz A, Rafolt D, Golaszewski S (2015). Modulation of sensorimotor cortex by repetitive peripheral magnetic stimulation. Front Hum Neurosci.

[17] Sasaki R, Nakagawa M, Tsuiki S, Miyaguchi S, Kojima S, Saito K, Onishi H (2017). Regulation of primary motor cortex excitability by repetitive passive finger movement frequency. Neuroscience.

[18] Kaelin-Lang A, Luft AR, Sawaki L, Burstein AH, Sohn YH, Cohen LG (2002). Modulation of human corticomotor excitability by somatosensory input. J Physiol.

[19] Ridding MC, Brouwer B, Miles TS, Pitcher JB, Thompson PD (2000). Changes in muscle responses to stimulation of the motor cortex induced by peripheral nerve stimulation in human subjects. Exp Brain Res.

[20] Struppler A, Havel P, Müller-Barna P (2003). Facilitation of skilled finger movements by repetitive peripheral magnetic stimulation (RPMS)–a new approach in central paresis. NeuroRehabilitation.

[21] Jiang YF, Zhang D, Zhang J, Hai H, Zhao YY, Ma YW (2022). A randomized controlled trial of repetitive peripheral magnetic stimulation applied in early subacute stroke: effects on severe upper-limb impairment. Clin Rehabil.

[22] Obayashi S, Takahashi R (2020). Repetitive peripheral magnetic stimulation improves severe upper limb paresis in early acute phase stroke survivors. NeuroRehabilitation.

[23] Fugl-Meyer AR, Jääskö L, Leyman I, Olsson S, Steglind S (1975). A method for evaluation of physical performance. Scand J Rehabil Med.

[24] Lyle RC (1981). A performance test for assessment of upper limb function in physical rehabilitation treatment and research. Int J Rehabil Res.

[25] Anarte-Lazo E, Rodriguez-Blanco C, Falla D, Bernal-Utrera. C. Musculoskeletal Science and Practice. 10.1016/j.msksp.2023.102738 36870148

[26] Qin Y, Liu X, Zhang Y, Wu J, Wang X (2023). Effects of transcranial combined with peripheral repetitive magnetic stimulation on limb spasticity and resting-state brain activity in stroke patients. Front Hum Neurosci.

[27] Wu X, Wang R, Wu Q, Liao C, Zhang J, Jiao H, Liu R. (2023). The effects of combined high-frequency repetitive transcranial magnetic stimulation and cervical nerve root magnetic stimulation on upper extremity motor recovery following stroke. Front NeuroSci, 17. 10.3389/fnins.2023.1100464 PMC9951778 36845428

[28] Zhang L, Xing G, Fan Y, Guo Z, Chen H, Mu Q (2017). Short-and long-term effects of repetitive transcranial magnetic stimulation on upper limb motor function after stroke: a systematic review and meta-analysis. Clin Rehabil.

[29] Bernhardt J, Hayward KS, Kwakkel G, Ward NS, Wolf SL, Borschmann K, Cramer SC (2017). Agreed definitions and a shared vision for new standards in stroke recovery research: the stroke recovery and rehabilitation roundtable taskforce. Int J Stroke.

[30] Brunnstrom S (1966). Motor testing procedures in hemiplegia: based on sequential recovery stages. Phys Ther.

[31] Wade DT, Wood VA, Hewer RL (1985). Recovery after stroke–the first 3 months. J Neurol Neurosurg Psychiatry.

[32] Ahdab R, Ayache SS, Brugières P, Goujon C, Lefaucheur JP (2010). Comparison of standard and navigated procedures of TMS coil positioning over motor, premotor and prefrontal targets in patients with chronic pain and depression. Neurophysiologie Clinique/Clinical Neurophysiol.

[33] Beaulieu LD, Masse-Alarie H, Brouwer B, Schneider C (2015). Noninvasive neurostimulation in chronic stroke: a double-blind randomized sham-controlled testing of clinical and corticomotor effects. Top Stroke Rehabil.

[34] RA K (1987). The functional independence measure; a new tool for rehabilitation. AdvClinRehabil.

[35] Mulder M, Nijland R (2016). Stroke impact scale. J Physiotherapy.

[36] Faul F, Erdfelder E, Buchner A, Lang AG (2009). Statistical power analyses using G* power 3.1: tests for correlation and regression analyses. Behav Res Methods.

[37] Cramer SC, Bastings EP (2000). Mapping clinically relevant plasticity after stroke. Neuropharmacology.

[38] Lin KC, Fu T, Wu CY, Wang YH, Liu JS, Hsieh CJ, Lin SF (2010). Minimal detectable change and clinically important difference of the stroke impact scale in stroke patients. Neurorehabilit Neural Repair.

[39] Huang YZ, Lu MK, Antal A, Classen J, Nitsche M, Ziemann U, Rothwell J (2017). Plasticity induced by non-invasive transcranial brain stimulation: a position paper. Clin Neurophysiol.

[40] Suppa A, Huang YZ, Funke K, Ridding MC, Cheeran B, Di Lazzaro V, Rothwell JC (2016). Ten years of theta burst stimulation in humans: established knowledge, unknowns and prospects. Brain Stimul.

[41] Mori F, Codecà C, Kusayanagi H, Monteleone F, Boffa L, Rimano A, Centonze D (2010). Effects of intermittent theta burst stimulation on spasticity in patients with multiple sclerosis. Eur J Neurol.

[42] Sala C, Piëch V, Wilson NR, Passafaro M, Liu G, Sheng M (2001). Regulation of dendritic spine morphology and synaptic function by Shank and Homer. Neuron.

[43] Chen YJ, Huang YZ, Chen CY, Chen CL, Chen HC, Wu CY, Chang TL (2019). Intermittent theta burst stimulation enhances upper limb motor function in patients with chronic stroke: a pilot randomized controlled trial. BMC Neurol.

[44] Kuzu Ö, Adiguzel E, Kesikburun S, Yaşar E, Yılmaz B (2021). The effect of sham controlled continuous theta burst stimulation and low frequency repetitive transcranial magnetic stimulation on upper extremity spasticity and functional recovery in chronic ischemic stroke patients. J Stroke Cerebrovasc Dis.

[45] Liu Y, Yin M, Luo J, Huang L, Zhang S, Pan C, Hu X (2020). Effects of transcranial magnetic stimulation on the performance of the activities of daily living and attention function after stroke: a randomized controlled trial. Clin Rehabil.

[46] Nyffeler T, Vanbellingen T, Kaufmann BC, Pflugshaupt T, Bauer D, Frey J, Cazzoli D (2019). Theta burst stimulation in neglect after stroke: functional outcome and response variability origins. Brain.

[47] Appelros P, Stegmayr B, Terént A (2009). Sex differences in stroke epidemiology: a systematic review. Stroke.

[48] VanGilder JL, Hooyman A, Peterson DS (2020). Post-stroke cognitive impairments and responsiveness to motor rehabilitation: a review.CurrPhys. Med Rehabil Rep.

